# Diagnostic performance of the molecular *BCR-ABL1* monitoring system may impact on inclusion of CML patients in stopping trials

**DOI:** 10.1371/journal.pone.0214305

**Published:** 2019-03-21

**Authors:** Birgit Spiess, Sébastien Rinaldetti, Nicole Naumann, Norbert Galuschek, Ute Kossak-Roth, Patrick Wuchter, Irina Tarnopolscaia, Diana Rose, Astghik Voskanyan, Alice Fabarius, Wolf-Karsten Hofmann, Susanne Saußele, Wolfgang Seifarth

**Affiliations:** 1 Department of Hematology and Oncology, University Hospital Mannheim, Heidelberg University, Mannheim, Germany; 2 Institute of Transfusion Medicine and Immunology, Medical Faculty Mannheim, Heidelberg University, Mannheim, Germany; SA Adelaide, AUSTRALIA

## Abstract

In chronic myeloid leukemia (CML), the duration of deep molecular response (MR) before treatment cessation (MR^4^ or deeper, corresponding to *BCR-ABL1* ≤ 0.01% on the International Scale (IS)) is considered as a prognostic factor for treatment free remission in stopping trials. MR level determination is dependent on the sensitivity of the monitoring technique. Here, we compared a newly established TaqMan (TM) and our so far routinely used LightCycler (LC) quantitative reverse transcription (qRT)-PCR systems for their ability to achieve the best possible sensitivity in *BCR-ABL1* monitoring. We have comparatively analyzed RNA samples from peripheral blood mononuclear cells of 92 randomly chosen patients with CML resembling major molecular remission (MMR) or better and of 128 CML patients after treatment cessation (EURO-SKI stopping trial). While our LC system utilized *ABL1*, the TM system is based on *GUSB* as reference gene. We observed 99% concordance with respect to achievement of MMR. However, we found that 34 of the 92 patients monitored by TM/*GUSB* were re-classified to the next inferior MR log level, especially when LC/*ABL1*-based results were borderline to thresholds. Thirteen patients *BCR-ABL1* negative in LC/*ABL1* became positive after TM/*GUSB* analysis. In the 128 patients included in the EURO-SKI trial identical molecular findings were achieved for 114 patients. However, 14 patients were re-classified to the next inferior log-level by the TM/*GUSB* combination. Eight of these patients relapsed after treatment cessation; two of them were re-classified from MR^4^ to MMR and therefore did not meet inclusion criteria anymore. In conclusion, we consider both methods as comparable and interchangeable in terms of achievement of MMR and of longitudinal evaluation of clinical courses. However, in LC/*ABL1* negative samples, slightly enhanced TM/*GUSB* sensitivity may lead to inferior classification of clinical samples in the context of TFR.

## Introduction

The occurrence of the reciprocal translocation between chromosomes 9 and 22 (t(9;22)(q34;q11)) resulting in a translocation of the genes *BCR* and *ABL1* is causal for development of chronic myeloid leukemia (CML).

The majority of patients express different *BCR-ABL*1 mRNA fusion variants (most commonly e13-a2, e14-a2, e1-a2) resulting in the expression of abnormal BCR-ABL1 fusion tyrosine kinase. Molecular diagnostics of CML and patient monitoring is based on quantification of BCR-ABL1 transcript levels in peripheral blood (PB) or bone marrow (BM) aspirates of patients by various qRT-PCR technologies as the number of *BCR-ABL1* transcripts serves as surrogate indicator for the amount of residual *BCR-ABL1* positive tumor cells and/or their proliferative potential during therapy with tyrosine kinase inhibitors (TKI). Therapy regimen employing TKI directed at the abnormal *BCR-ABL1* fusion tyrosine kinase can achieve durable cytogenetic and molecular remissions (MRs) and improve survival in the majority of patients which is approaching that of the general population [1–3]. Since a high percentage of the patients achieve deep molecular response (DMR) under TKI treatment the conception of treatment free remission (TFR) was supported [4–6]. TKI treatment has been stopped successfully in approximately half of the patients with DMR in cessation trials [7, 8]. The criteria for discontinuation differ in the various studies [9]. Most have defined MR^4^ or MR^4.5^ for at least one year and TKI treatment duration of at least three years [7]. Currently, the European EURO-SKI trial evaluating molecular data of 755 patients after TKI discontinuation is the first study to define the best criteria for cessation of TKI treatment in patients with CML in deep molecular response [10]. The interim multivariable analysis revealed that the probability of MMR maintenance was significantly associated with overall TKI treatment duration, interferon pre-treatment, and DMR duration, the latter showing the largest effect on the success of treatment cessation [10]. 373/755 (49%) patients analyzed so far in EURO-SKI trial have lost MMR after 24 months of TKI cessation.

Achieving a TFR is desirable, as it reduces treatment costs and avoids therapy-associated side effects and late sequelae of therapy. So far, it is unclear how deep the MR should be before a stopping attempt. In this context, the log reduction of detectable *BCR-ABL1* fusion transcripts, is a crucial factor in the decision to TKI discontinuation and essentially depends on the definition of molecular response as well as on technical aspects of *BCR-ABL1* transcript measurements. Therefore, a highly standardized assay system with the highest possible sensitivity has to be considered the most appropriate test system [11].

New revised definitions were introduced by the CML Working Group of the ELN that take into account the sensitivity of the molecular qRT-PCR test, that is, MR^4^ indicates ≥ 4-log reduction (BCR-ABL IS ≤ 0.01%), MR^4.5^ indicates ≥ 4.5-log reduction (BCR-ABL IS ≤ 0.0032%), and MR^5^ indicates ≥ 5-log reduction (BCR-ABL IS ≤ 0.001%), especially in negative qRT-PCR results. Mandatory for valid calculation of major responses (log reduction) is a sufficient RNA quality as given by the absolute copy numbers of housekeeping genes *ABL1* and/or *GUSB* [11, 12]. qRT-PCR using LightCycler (LC, Roche Applied Science, Mannheim, Germany) and TaqMan (TM; ThermoFisher Scientific/Applied Biosystems, Darmstadt, Germany) technologies meet all the requirements for sensitive and reliable diagnostic tools to perform molecular monitoring in CML patients [13–15]. The well-established LC methodology and newly implemented TM system are routinously used in our laboratory, standardized according to IS [16, 17], in strict consent with the international guidelines [18, 19]. The high fidelity and longitudinal reproducibility of our measurements are granted by a stringent intramural quality and validation management [20].

Here, we report on the development of a novel protocol for *BCR-ABL1* monitoring using TM/GUSB technology. Moreover we present data on the *BCR-ABL1* monitoring of 220 CML patients (92 and 128 patients) with MMR or better comparatively tested with TM and LC. We evaluated accomplishment of the best possible sensitivity in *BCR-ABL1* transcript level assaying and we discuss the impact of our results in terms of involvement of patients in TKI stopping trials.

## Materials and methods

### Clinical sample preparation and controls

For RNA extraction, the clinical samples were processed using the automated Maxwell MDx technology (Promega, Mannheim, Germany). A PB sample of a healthy person processed in parallel to patients’ samples served as negative control for each processing cycle. In case of false positivity the qRT-PCR data of all corresponding (processed in parallel) clinical samples was rejected and RNA was prepared again employing frozen backup material. cDNA synthesis was performed using random hexamers and Superscript II reverse transcriptase (ThermoFisher Scientific, Darmstadt, Germany) as described previously [13].

The serial diluted standard plasmid provided an external positive control for each LC run. A PCR mix control served as negative control for each PCR experiment. For quality control of the sample material the internal reference transcript of *ABL1* or *GUSB* of each sample was used. All controls and samples were performed in duplicate. Assay stability and longitudinal performance of the qRT-PCR assays were assessed by the use of quality control charts (QCC) as described previously [20].

### qRT- PCR on LC platform

The LC PCR and detection system (version 2, Roche Applied Science) was used for amplification and quantification of *ABL1* control and *BCR-ABL1* fusion genes. The PCR reactions were performed in glass capillaries employing a LC “Fast Start DNA Master Hybridization Probes” kit (Roche Applied Science), *BCR* and *ABL1* specific primer and fluorescent probes as described by Emig et al., 1999 [13]. qRT-PCR for *BCR-ABL1* and *ABL1* transcripts was performed in duplicates using cDNA of diagnostic specimens prepared according to a standardized operating procedure (SOP), (Emig et al., 1999), and of seven serial dilutions of 4, 10, 40, 400, 4000, 40,000 and 400,000 copies of standard plasmid pME-2 per reaction [21]. The limit of detection (95%) (LOD_95_) is 4 copies using serial dilutions of pME-2.

For a LC experiment to be acceptable the generated data has to meet the passing criteria described in detail by Cross et al. [11]. The tolerable difference of the two single measurements of the standard and sample duplicates were evaluated according to Foroni et al., 2011 [19].

### qRT- PCR on TM platform

The TM PCR detection system (TaqMan 7500 Fast Real-Time PCR System, ThermoFisherScientific/Applied Biosystems) was used for amplification and quantification of *GUSB* control and *BCR-ABL1* fusion genes (e13-a2 and e14-a2). qRT-PCR for *BCR-ABL1* and *ABL1* transcripts was performed in 96-well plates in duplicates using cDNA of diagnostic specimens and of eight serial dilutions of 3, 15, 30, 300, 3000, 30,000 and 300,000 and 3 x 10^6^ copies of commercially available standard plasmid pIRMM0099 (ERM-AD623/”Institute for Reference Materials and Measurements”) per reaction. Available TM primers were for *BCR-ABL1*: ENF 501 (sense) 5’-TCCGCTGACCATCAAYAAGGA-3’ and ENR 561 (antisense) 5’-CACTCAGACCCTGAGGCTCAA-3’ [15, 22]. The expected sizes of *BCR-ABL1* amplicons for b2a2 and b3a2 fusion points transcripts are 228 bp and 153 bp in length, repectively. As *BCR-ABL1* specific probe ENP541-MGB sense (6FAM-CCCTTCAGCGGCCAGT-MGB) was used. *GUSB* specific primers were ENF 1102 (sense) 5’-GAAAATATGTGGTTGGAGAGCTCATT-3’ and ENR1162 (antisense) 5’-CCGAGTGAAGATCCCCTTTTTA-3’. The *GUSB* fragment is 101 bp in length. For detection of *GUSB* the specific probe ENPr1142 (antisense)-MGB was used (NED-CCAGCACTCTCGTCGGTGACTGTTCA-MGB). The specific primer pair for *ABL1* was: ENF 1003 (sense) 5’-TGGAGATAACACTCTAAGCATAACTAAACCT-3’ and ENR 1062 (antisense) 5’-GATGTAGTTGCTTGGGACCCA-3’. The PCR fragment is 124 bp long; the *ABL1* specific probe was ABL-1043V-MGB antisense (VIC-CATTTTTGGTTTGGGCTTC-MGB). For *BCR-ABL1* detection FAM was used as reporter dye and NFQ-MGB as quencher, for *GUSB* the reporter dye was NED, NFQ-MGB was used as quencher, *ABL1* was detected with the reporter dye VIC, as quencher NFQ-MGB was used, too. Primers and probes were used in a final concentration of 10 μM per PCR reaction. The amplification was performed in a total volume of 20 μl per reaction, whereby *BCR-ABL1* and *GUSB* amplification was performed as duplex PCR in one well. For each reaction 10 μl of a TaqMan Fast Advances Master Mix (ThermoFisherScientific/Applied Biosystems) were added. The PCR program implied at first a holding stage at 50°C for 2 min, than an additional holding stage at 95°C for 20 s, the cycling stage comprised 95°C for 3 s and 60°C for 45 s, the cycling stage was repeated for 45 times.

Criteria for passing for each TM run were: determination of gene specific different threshold values which were for *GUSB* = 0.06, for *ABL1* = 0.08 and for BCR-*ABL1* = 0.1. Achievements of further technical values were prerequisites: R^2^ > 0.98, Slope = -3.2 to -3.6 and intercept ≤ 42. At least four standards of the standard series have to be evaluable, whereby at least for *BCR-ABL1* measurements one duplicate of standard 3 or standard 15 has to be positive. For *GUSB* and *ABL1* at least one of the standard 300 duplicates has to be positive. All duplicates were evaluated according to the guidelines of Foroni., 2011 [19]. The cut off for the evaluation of positive signals from negative control blood samples and patients’ samples was Ct = 42. Housekeeping genes and internal references *ABL1* and *GUSB* are commonly used. For MMR, MR^4^, MR^4.5^ and MR^5^, at least 24,000; 24,000; 77,000 and 240,000 *GUSB* copies, or 10,000; 10,000; 32,000 and 100,000 *ABL1* copies are necessary, respectively [11].

### Quality management for qRT-PCR data

Ct values (Cycle threshold) of the standard dilution series of the respective qRT-PCR experiments (LC and TM) were exported into a Microsoft Excel sheet. These data was compared to the mean values and the respective warning and control (intervention) limits given by the respective QCC currently valid. Standard dilution data for each experiment must meet QCC requirements for passing. The analysis and documentation of the Ct values and the comparison to warning and control (intervention) limits is done automated by an algorithm implemented in our proprietary lab information management software (LIMS). Exceeding the limits is documented by our LIMS database (LeukoDB2) [20].

### Statistics

Statistical calculations were performed using Microsoft Excel and GraphPad Prism (V 6.0) according to standard procedures. We have applied simple column statistics (mean, median, range). In addition unpaired (T-test) and paired analysis (Wilcoxon signed rank test) were employed for calculation of variation ranges between LC/*ABL1* and TM/*GUSB* analysis.

### Ethical considerations

Control blood samples of healthy donors were obtained with written informed consent in accordance with the declaration of Helsinki from the local blood bank in fully anonymized manner.

Analyses were done according to Good Clinical Practice (GCP) guidelines as well as in concordance with the Declaration of Helsinki. The investigations were approved by the local Ethics Committee (Medizinische Ethikkommision II der Medizinischen Fakultät Mannheim der Ruprecht Karls-Universität Heidelberg, 2013-509N-MA and 212-247-AWB-MA). This analysis included 128 CML patients from the European Stop Tyrosine Kinase Inhibitor Study (EURO-SKI) trial, a prospective multicenter TKI discontinuation trial (NCT01596114). In accordance with the Declaration of Helsinki, written informed consent was obtained from all patients.

## Results

### Establishment and validation of TM PCR methodology

For establishing and implementing the TM technology, various parameters of the technology were tested within our quality management as part of method validation [23].

These were:

- Inter and intra experimental accuracy
- Robustness
- Limit of detection (LOD_95_)

### (i) Inter and intra experimental accuracy

**Fig 1:**
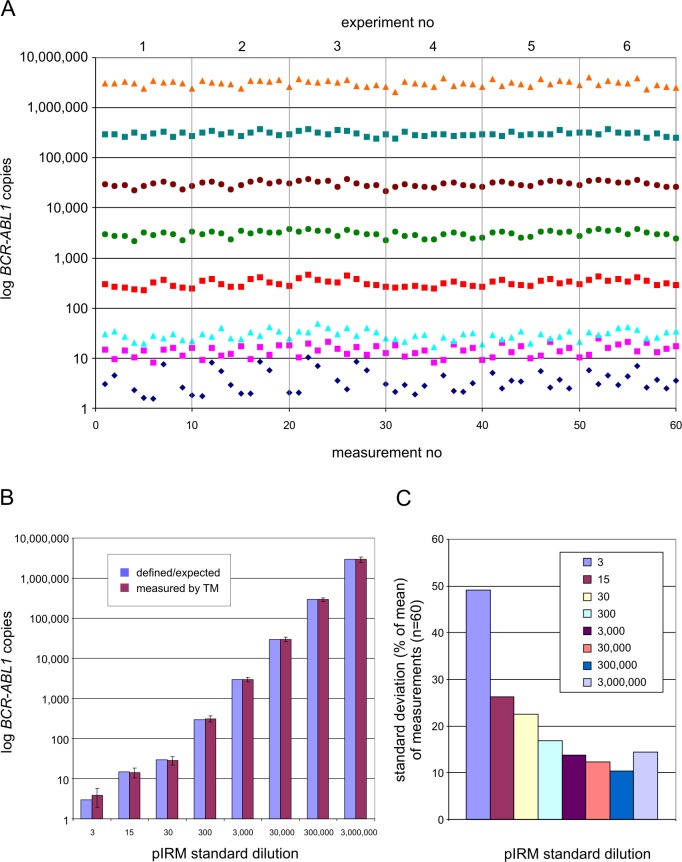
Diagnostic accuracy. Assessment of inter- and intraexperimental accuracy by TM PCR using pIRM standard dilutions representing 3–3,000,000 plasmid molecules per assay. (A) Six independent experiments with 10 replicates each revealed 60 consecutive data points for each of the 8 standard dilutions as depicted by the symbols in horizontal order. (B) Calculated variance of standard dilution measurements with regard to the defined/expected plasmid molecule numbers as theoretically deployed in the assay. (C) Standard deviation (in % of mean) of measurements shown in (A).

For determination of the accuracy, six experiments were performed in parallel using the standard series (pIRM 3–3,000,000), generating a total of 60 data points and 10 wells to quantify *ABL1*. Results are shown graphically in Fig 1A–1C.

The acceptance criteria were met after evaluation of the data. The correctness was given because the device software was able to generate a calibration straight line with R^2^ > 0.98 [19] for each of the six evaluations and the standard deviations (%) were not greater than the permitted fluctuations (within warning and control limits) [20]. Naturally, the standard deviation increases with decreasing number of molecules in the sample (standards 3, 15, 30) as the influence of pipetting inaccuracy increases. For standard 3, the mean for the measured real value was 3.9 *BCR-ABL1* copies (+/- 1.95 (49%)), for standard 15, 14.2 copies (+/- 3.77 (26%)), for standard 30, 28.6 copies (+/- 6.56 (23%)), for standard 300, 315 copies (+/- 54 (17%)), for standard 3,000, 3,015 copies (+/- 423 (14%)), for standard 30,000, 29,563 copies (+/- 3,698 (12%)), for standard 300,000, 292,894 copies (+/- 31,350 (10%)) and mean for standard 3,000,000 was 2,943,016 copies (+/- 436,236 (14%)) (Fig 1B and 1C).

### (ii) Robustness

A diagnostic method can be considered robust if the same results are obtained even though slight variations (operator- or hardware-related) in the experimental procedure may have occurred. To test the TM PCR for robustness, five independent experiments were performed using the plasmid standard dilution series (pIRM 3–3,000,000) by varying the following parameters:

- Altered annealing temperature: the assay was carried out by one single operator but at varying annealing temperatures (1°C below and 1°C above the standard annealing temperature of 60°C).
- Variations by different operator: two identical standard experiments were performed in parallel by two different lab operators.
- Altered volume of master mix added to PCR reaction: from the master mix only 16 μl were used instead of 17 μl, so the PCR reaction was carried out in 1 μl less volume than according to standard protocol (= 17 μl master mix + 3 μl cDNA sample).

**Fig 2:**
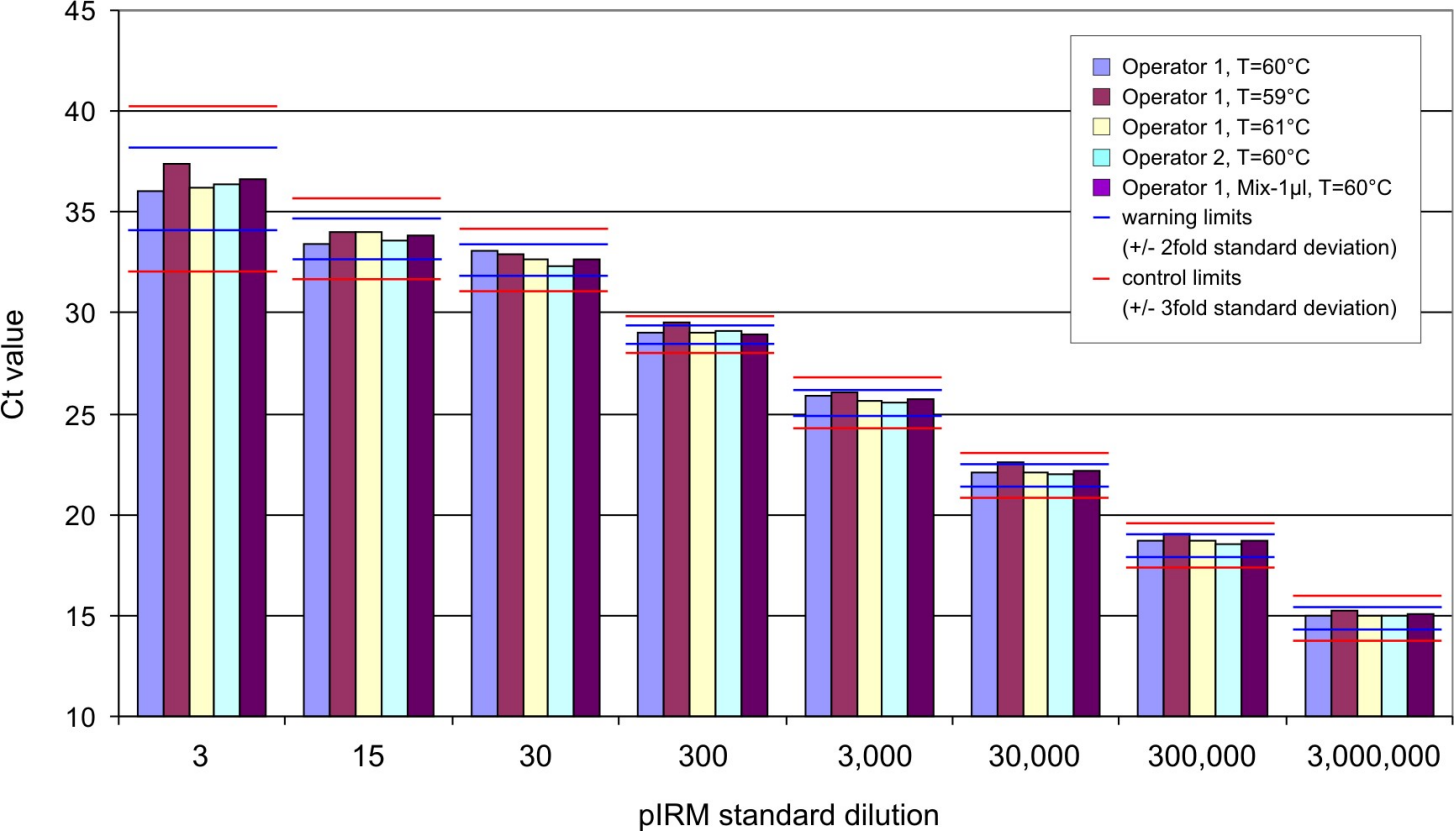
Determination of robustness of TM methodology using the plasmid standard series pIRM. Annealing temperatures (59°C, 60°C, 61°C), operators (n = 2) and volumes of master mix added to PCR template were varied. All experiments were performed in duplicates. Blue and red lines above data columns depict warning (+/- 2fold standard deviation) and control limits (+/- 3fold standard deviation), respectively.

The Ct values output by the TM software were compared to the Ct values of the intra-experimental variation (IEV = upper and lower warning limits according to QCC) [20]. For robustness, the values should be within the range specified as warning limits (= average values +/- 2 x standard deviation). As shown in Fig 2 this was achieved in the majority of experiments. Only at the standard dilutions 300 and 30.000 the upper warning limit was hit when the annealing temperature was decreased by 1° C (59° C instead of 60° C). The values were still below the control limit (+/- 3 x standard deviation) and therefore within the inter-experimentally acceptable variation.

### (iii) Limit of detection (LOD_95_)

For reliable detection of low target molecule numbers the assay validation should include the determination of the assay’s detection limit (LOD_95_). LOD_95_ represents the number of target molecules that will be reliably detected in 95% of all tested samples. For calculation of LOD_95_ for the TM-based qRT-PCR the data of 40 consecutive measurements of standard dilution series (pIRM 3–3,000,000) were evaluated. For standard dilution 3 and 15 the expected signals were positively detected in 38 and 40 cases corresponding to 95% and 100% of detection, respectively. The detection limit of LOD_95_ was thus 3 molecules in the sample. The detection limit in the diagnostic system can be considered < 4 target molecules in the reaction mixture, which can be rated as good. It is in line with the internationally achievable sensitivity standards for TM and LC technology [11].

### Comparison of LC results to TM results

For the comparative assessment of the *BCR-ABL1* quantification by means of the TM and LC method with regard to the reliable achievement of the diagnostic statement "achievement of MMR—yes / no", 92 patient consecutive samples of daily routine diagnostic operation were analyzed in parallel using both qRT-PCR technologies. The calculated values are based on the means of duplex experiments. A comparison of the findings produced in parallel with LC and TM revealed the following similarities and changes of log levels (Table 1) including 16 of 19 (TM/*GUSB*) and 11 of 19 (TM/*ABL1*) patients who lost their MR^5^ status. Thirteen of 16 patients with formerly undetected *BCR-ABL1* transcripts were analyzed positive using TM/*GUSB*. Four cases changed from MR^5^ to MR^4.5^, three, four and two cases changed from MR^5^ to MR^4.0^, MR^4.5^ to MR^4^ and from MR^4.5^ to MMR, respectively.

**Table 1:** TM and LC findings in comparison.

Methods	Identical findings	Log changes MR^5^ to MR^4.5^ or MR^4^	Log changes MR^4.5^ to MR^4^ or MMR	Log changes MR^4^ to MMR	Other log changes
LC/*ABL1* vs TM/*GUSB*	58/92 (63%)	16 (17%)	9 (10%)	7 (8%)	2 (2%)
LC/*ABL1* vs TM/*ABL1*	62/92 (70%)	11 (12%)	8 (9%)	5 (5%)	6 (7%)

**Fig 3:**
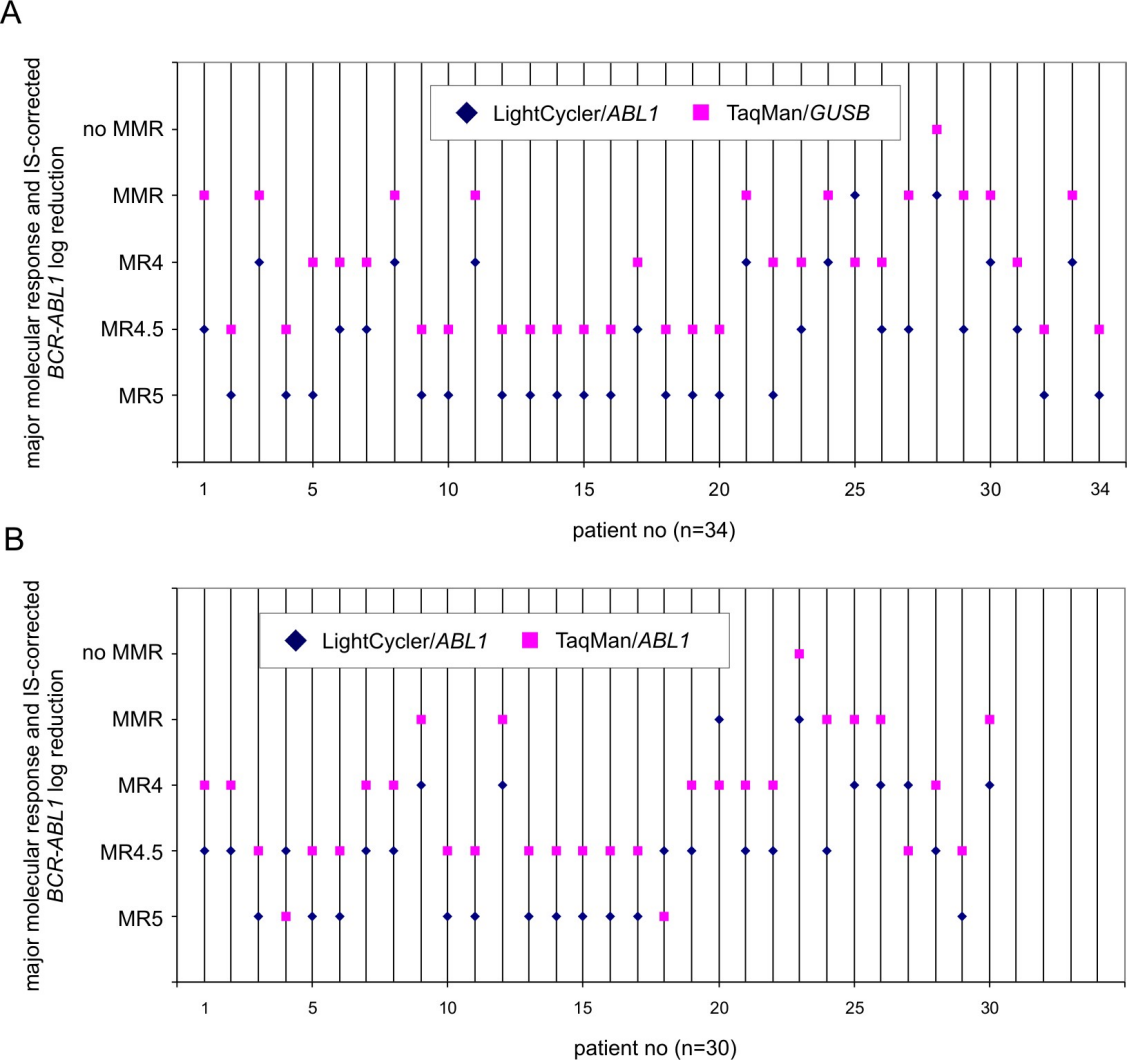
Diagnostic outcome. **A.** Assay-related shifts (n = 34) in the diagnostic outcome of 92 CML patient samples were analyzed with the TM/*GUSB* combination (pink squares) instead of LC/*ABL1* combination (blue diamonds). **B.** Assay-related shifts (n = 30) in the diagnostic outcome of 92 CML patient samples were analyzed with the TM/*ABL1* combination (pink squares) instead of LC/*ABL1* combination (blue diamonds). Abbreviations: MMR, major molecular response; MR^4^ indicates ≤ 4-log reduction (BCR-ABL1 IS ≤ 0.01%), MR^4.5^ indicates ≤ 4.5-log reduction (BCR-ABL1 IS ≤ 0.0032%), and MR^5^ indicates ≤ 5-log reduction (BCR-ABL1 IS ≤ 0.001%). The prerequisite for valid calculation of major responses (log reduction) is a sufficient RNA quality as given by the absolute copy numbers of housekeeping gene *GUSB*.

The differences in diagnostic findings for *BCR-ABL1* monitoring between LC using *ABL1* and TM using *ABL1*/*GUSB* as housekeeping genes is graphically shown in Fig 3.

We have calculated the variation ranges of LC/*ABL1* (mean: 0.003; median: 0.00; SEM: 0.0085; range: 0 to 0.047) versus TM/*GUSB* (mean: 0.011; median: 0.004; SEM: 0.027; range: 0 to 0.157) using the Wilcoxon signed rank test (p<0.0001) based on those samples where changes in log levels have been found (n = 34).

All clinical course data before January 2018 was LC/ABL1 based. Since then, TM/GUSB was used for *BCR-ABL1* patient monitoring. After evaluation of the overall clinical courses of all 92 patients under consideration of LC/*ABL1* and the available TM/*GUSB* data, it turned out that single TM/*GUSB* data points have no impact on the overall clinical course. However, we found slightly enhanced sensitivity for the TM/*GUSB* combination. This is statistically valid as pairwise testing (Wilcoxon signed rank test) revealed p<0.0001 when comparing the 92 data points of LC/*ABL1* (mean 1.828 +/- 0.9983, range 0.00 to 58.00) to the corresponding 92 data points of TM/*GUSB* (mean 1.901 +/- 1.078, range 0.00 to 82.75). In about 30% of patients, this led to inferior staging as exemplarily shown for eight patients (#1 to #8) in S1 Fig. The differences in sensitivity between LC/*ABL1* and TM/*GUSB* measurements become clearer the more TM/*GUSB* data points were available as depicted in the clinical courses of patients (#4 to #8). Here, a clear sensitivity shift can be observed nevertheless concurring with the respective trends of the clinical courses established by the preceding LC/*ABL1* data points. The enhanced sensitivity of TM/GUSB is reflected by the fact that many sample points that were negative in LC/*ABL1* analysis became positive when TM/*GUSB* method was applied (patients #1, 2, 5, 6, 8). This phenomenon is independent of the conversion factor (CF) applied for calculation of the quotient *BCR-ABL1* IS [%].

### Re-evaluation of EURO-SKI samples using TM technology

**Fig 4:**
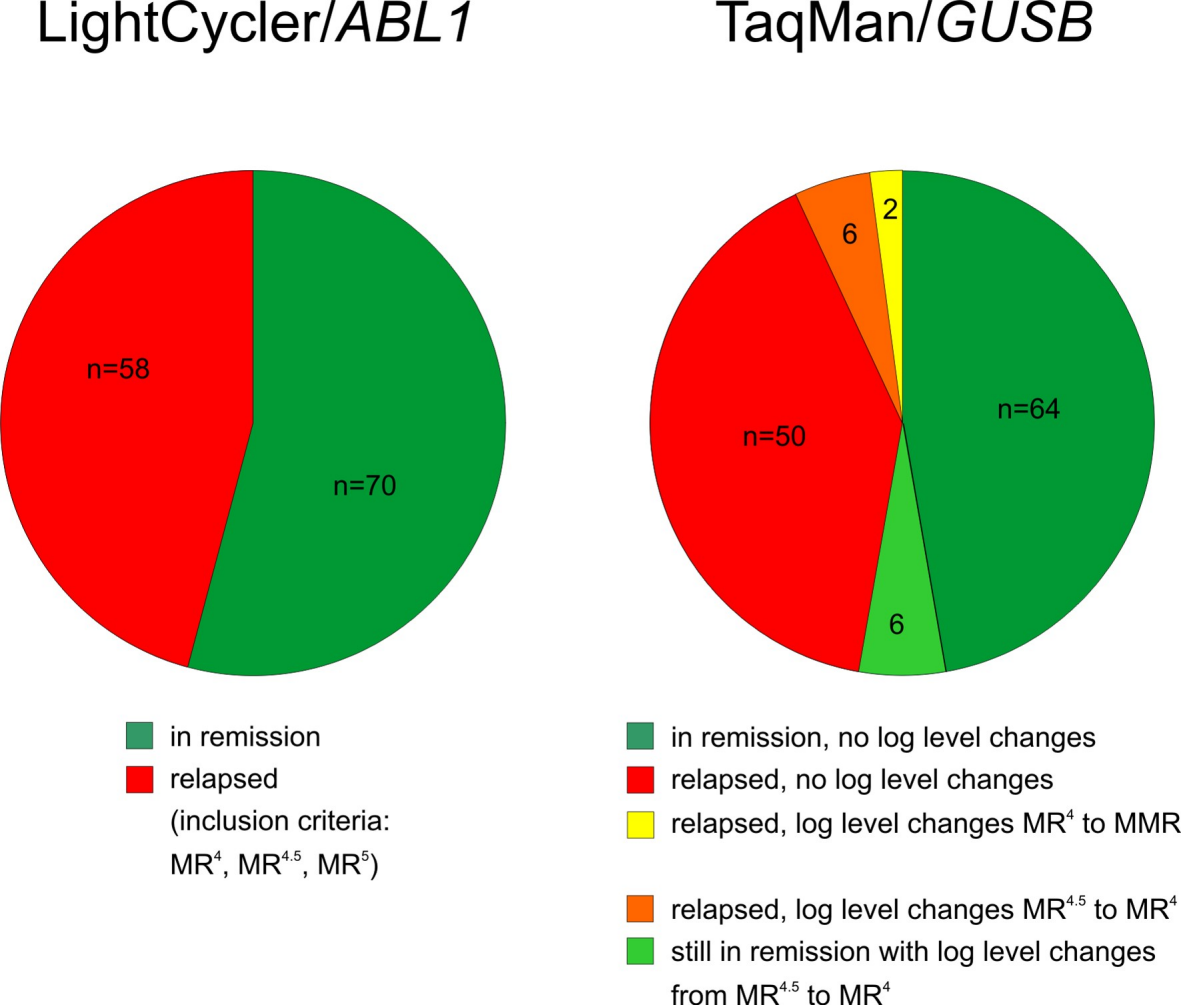
EURO-SKI samples comparatively tested with LC/*ABL1* and TM/*GUSB* system. Comparison of EURO-SKI stopping trial interim TFR analysis data (left pie) with a “what if” scenario (right pie) where patient inclusion was based on more sensitive TM/*GUSB* instead of LC/*ABL1* assays. Of 128 patients who were evaluated by LC/*ABL1* (left pie), 58 (45%) relapsed within 24 months of TKI discontinuation. According to data based on TM/*GUSB* monitoring of 128 EURO-SKI patients (right pie), two of the relapsing patients (2/58 = 3.4%) were re-classified from MR^4^ to MMR and, therefore, both specimen analyzed did not meet the inclusion criteria anymore (yellow sector). The same holds true for another 6 patients (6/58 = 10%) when MR^4^ was excluded as inclusion criterion (orange sector). Six samples of patients in remission were re-classified from MR^4.5^ to MR^4^ but are currently still in TFR (light green sector).

In order to assess the potential impact of the higher sensitive TM/*GUSB* combination on inclusion of patients in stopping trials we performed a “what if” analysis on a panel of 128 patients of the EURO-SKI stopping trial that have been previously monitored by employment of the LC/*ABL1* combination. One sample of each patient representative of the screening period before inclusion in the stopping trial (i.e. MR^4^ mandatory for at least one year) was included in the analysis. Identical results were obtained for 114 patients irrespective whether LC/*ABL1* or TM/GUSB was used for monitoring concerning MR^4^ in four and MR^4.5^/MR^5^ in 110 patients. Fourteen patients were re-classified to the next inferior log-level when using the TM/*GUSB* system. Altogether 12 patients were re-classified from MR^4.5^ to MR^4^. Of these 12, six patients showed relapse and the other six are still in TFR but are potential candidates for relapse and are under special attention. Upon hypothetic application of our “what if” scenario, the six relapsing patients were “saved” when inclusion into the stopping trial was restricted to MR^5^ and MR^4.5^ patients. Two patients of the relapse group were re-classified from MR^4^ to MMR and would not have led to cessation of treatment (Fig 4).

We have added the variation ranges of actual *BCR-ABL1* quotient IS [%] ratio for the 14 patients who were diagnosed differently when LC/*ABL1* and TM/*GUSB* techniques were applied. As LC/*ABL1* showed a mean variation of 0.0027 (+/- 0.0021) (range: 0.00097 to 0.0097) whereas the TM/*GUSB* displayed a mean variation of 0.006 (+/- 0.0035) (range 0 to 0.0144). Wilcoxon signed rank test revealed significance (p = 0.0023) between both groups.

## Discussion

We demonstrated that our newly established *TM/GUSB* system is a robust and reliable qPCR method for monitoring CML patients. The TM technology for *BCR-ABL1* quantitative measurements in our laboratory using the diagnostic combination *TM/GUSB* improved the sensitivity when compared to *LC/ABL1* technology [13]. We found that 34 (37%) and 30 (33%) of the 92 patients monitored by TM/*GUSB* and TM/ABL1, respectively, were re-classified to the next inferior log level when compared to LC/*ABL1* results. This includes 16 of 19 patients who lost their MR^5^ status in TM/*GUSB* analysis, indicating that some (13 of 92) of the “*BCR-ABL1*-negative” samples tested may not be really negative if higher sensitive methods are applied. Albeit the slightly observed enhanced sensitivity of TM/*GUSB* that in some cases may lead to inferior classification of single data points, we consider both methods as absolutely comparable and interchangeable in terms of evaluating longitudinal clinical courses (compare S1 Fig).

This The observed enhanced sensitivity of TM/*GUSB* is important, as one could consider that employment of more sensitive diagnostic methods could push the limits for inclusion of patients in stopping trials and finally may reduce the number of patients incurring loss of MMR after TKI discontinuation. Therefore, it seems mandatory to use the most efficient logistics and most sensitive techniques for sample collection and transportation, RNA preparation and qRT-PCR.

In fact, when comparing both PCR systems and generating a “what if” scenario for retrospectively selected cDNA samples of 128 EURO-SKI patients identical results were obtained only for 114 patients concerning MR^4^ in four and MR^4.5^/MR^5^ in 110 patients. TM/*GUSB* PCR findings were different from LC/*ABL1* findings in 14 patients. Of these, eight patients were from the relapse group; in two of these patients the TM/*GUSB* PCR results would not have led to cessation of therapy, suggesting that an employment of more sensitive PCR systems may spare patients disease progression. It is conceivable that patients that are borderline to threshold ranges may shift to the next inferior log level (MR^4^ -> MMR) and therefore lose their inclusion criterion.

Six of the 14 patients re-classified from MR^4.5^ to MR^4^ had no relapse during TFR in a follow up period of at least 24 month [10]. Further monitoring will show future loss or maintenance of TFR in these patients. There could be manifold reasons for higher TM/*GUSB* qRT-PCR sensitivity. In the TM/*GUSB* PCR assay, there are differences in primer combinations and generated PCR amplicons. In determining the sensitivity of a PCR assay, primer selection and the length of the resulting PCR amplicons may play a crucial role. Shorter PCR fragments (length: 100–150 bp) are amplified in the TM system with higher efficiency. Furthermore TM and LC are different PCR systems with different PCR performance and kinetics. The TM technology uses only one labelled probe as a 5’ nuclease assay to generate a specific fluorescence signal instead of two probes, resulting in a simplification of the PCR kinetics which contributes to an increase in sensitivity. In addition, another type of Taq Polymerase used in TM technology could also influence PCR sensitivity and efficiency. Also the choice of the housekeeping gene may influence the diagnostic outcome. After careful testing and evaluating so far published data, we prefer *GUSB* as housekeeping gene for TM-based analysis. While Beillard et al. [24] recommended *ABL1* as control gene for RQ-PCR-based diagnosis and MRD detection in leukemic patients because of more stable, uniform expression and lack of pseudogenes, *GUSB* transcript levels were later confirmed as a suitable alternative [25]. Other authors described the suitability of *ABL1*, *BCR* and *GUSB* for *BCR-ABL1* quantification [15] or even considered beta-glucoronidase (*GUSB*) as the most suitable genes tested [26] because the expression pattern of beta-glucoronidase (*GUSB*) was found more homogenous than that of *ABL1* or β2 microglobulin (*B2M*) [27]. Not to be neglected the main practical advantage of the use *GUSB* over *ABL*1 as housekeeping genes is the performance of *BCR-ABL1* and *GUSB* quantification in one single well (duplex PCR) avoiding the bias introduced by setting-up two spatially distinct TM qPCR reactions.

One could argue that the observed shift in log levels may purely reflect the variability of inter or intra-experimental variation or just pipetting inaccuracy, naturally observed for every assay system in particular when testing was performed for very low numbers of target molecules (compare Fig 1C). This may be the case for samples in log levels MR^4.5^ and MR^5^ where very few numbers (<10) of *BCR-ABL1* cDNA molecules decide on sample positivity. This becomes evident in clinical courses of some representative patients shown in S1 Fig, where positivity and negativity fluctuates in consecutive measurements. These fluctuations seem to be normal and do not impact the overall clinical courses as depicted in S1 Fig but may be important in TFR-related settings. However this variation is expected to follow a random Poisson distribution as described previously [20]. In contrast, in our methodological comparison the majority of samples increased in log levels pointing to a non-random change in assay sensitivity (= assay variation).

One could further argue that the increased sensitivity of TM/*GUSB* versus LC/*ABL1* is merely due to an incorrect CF that may lead to erroneously high *BCR-ABL1* IS quotients. Our CFs were established using Bland Altman method by the Australian laboratory of Susan Branford and/or the EUTOS consortium. However, the detection of *BCR-ABL1* copies in 13 cases where LC/*ABL1* did not succeed in detecting residual *BCR-ABL1* transcripts (0.000% IS) is independent of the CF and therefore, our data point to higher sensitivity of TM/*GUSB* when compared to LC/*ABL1*. Therefore it seems not advantageous to us to change the CF by own calculation. The clinical relevance of the detection of minimal amounts of *BCR-ABL1* copies using a more sensitive PCR system remains to be unclear and could be discussed controversially. Our preliminary data are a hint that increased sensitivity of the PCR system could play an important role in the scenario of stopping trials as being the basis for determination of MR^4^ and MR^4.5^ and therefore for the decision of treatment discontinuation. The duration and the log range of molecular remission is the factor with the highest predictive value for deciding on discontinuation of therapy. Therefore, the methods used to detect molecular remission play a crucial role. The deepening of the sensitivity of the diagnostic technologies depends on many different methodological factors. The quality of the RNA and the efficiency of the cDNA synthesis have an influence on the detected copy numbers of housekeeping gene and *BCR-ABL1*. A more efficient cDNA synthesis, together with a more sensitive PCR system, provides higher absolute copy numbers of *BCR-ABL1* and housekeeping gene, which have an influence on the generation of the respective log level for the individual patient.

The increased sensitivity of the TM/*GUSB* diagnostic system resulted in detection of *BCR-ABL1* copies in 13 cases where LC/*ABL1* did not succeed in detecting residual *BCR-ABL1* transcripts (0.000% IS). On the other hand, more sensitive systems generate the possibility to detect very high amounts of housekeeping gene copy numbers creating log level MR^5.5^ or even MR^6^, when a patient is in fact *BCR-ABL1* negative by qRT-PCR. It should be emphasized that inclusion criteria should grant the lowest number of relapsing patients at long sight. Aiming at this, it is conceivable that omitting the MR^4^ log level as inclusion criterion may be advantageous for the outcome of future stopping trials.

In conclusion, although enhanced sensitivity of TM/*GUSB* combination may be advantages the *BCR-ABL1* monitoring in some cases we consider both methods as comparable and interchangeable in terms of MMR achievement and evaluation of clinical courses. However, in LC/*ABL1* negative samples, slightly enhanced TM/*GUSB* sensitivity may lead to positivity and inferior classification of clinical samples.

Our data suggests that in clinical terms the diagnostic discrimination of MR^5^ and MR^4.5^ *BCR-ABL1* log levels may be clinically less important for the outcome of a stopping trial than the discrimination between MR^4.5^ and MR^4^, since the latter is nearby MMR. Our study is not meant to give statements about “correct” and “incorrect” methods, but is intended to serve optimization of molecular methods in terms of TFR-related settings.

The clinical impact of resulting changes needs to be further supported in large scale studies within stopping trials. We believe that the development of higher sensitive detection methods will positively impact on diagnostics and treatment of CML patients.

## Supporting information

S1 Fig Evaluation of longitudinal clinical courses of eight patients with CML. (TIF)
