# Supplementary figures and images for: Diagnostic performance of the molecular BCR-ABL1 monitoring system may impact on inclusion of CML patients in stopping trials
Source: PLoS One. 2019 Mar 21;14(3):e0214305. doi: 10.1371/journal.pone.0214305 (PMC6428315; doi:10.1371/journal.pone.0214305)

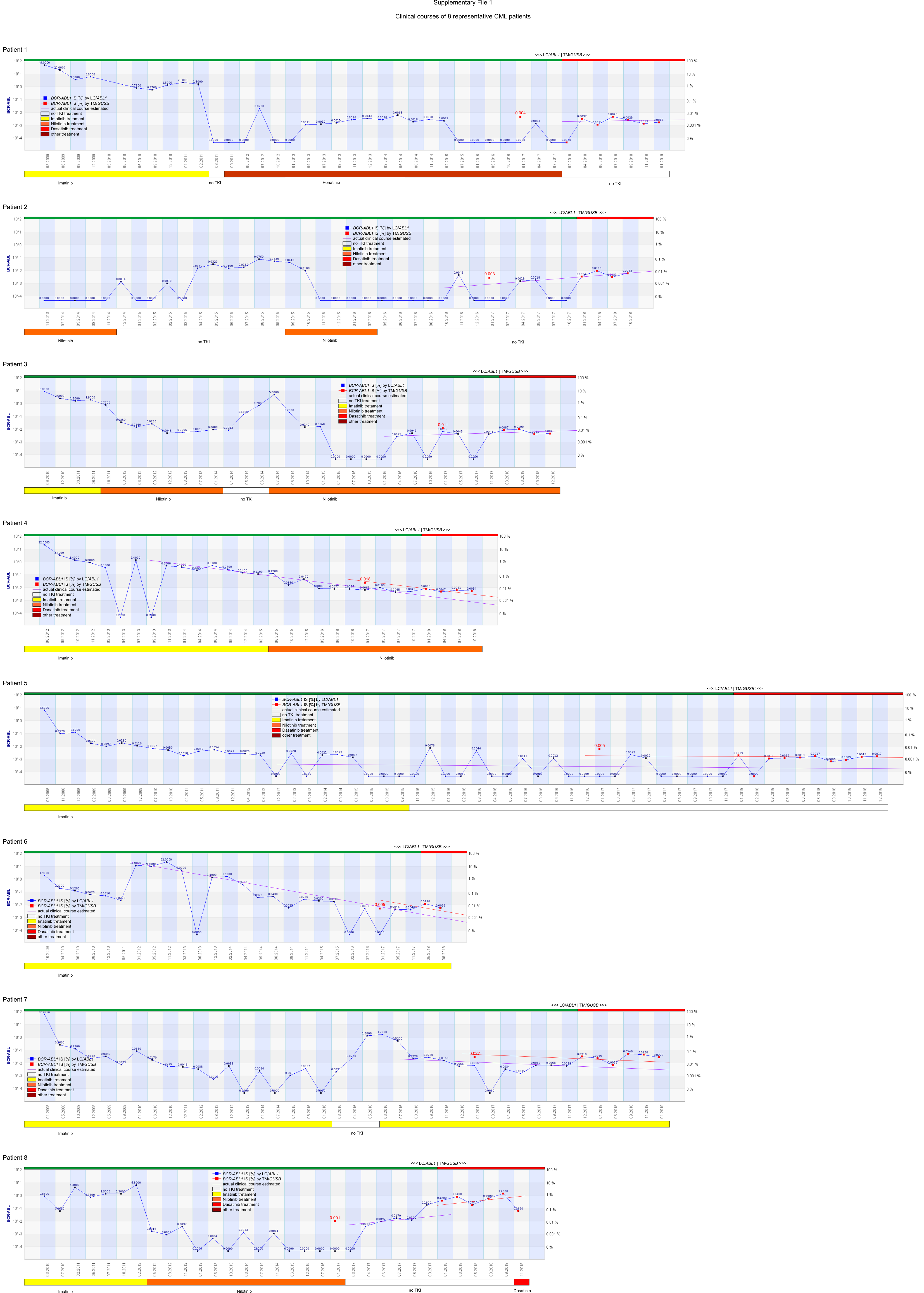

Supplement: S1 Fig — (TIF) [file pone.0214305.s001.tif]
